# The Methylation of Clock Genes in Perinatal Depression: Which Role for Oxytocin?

**DOI:** 10.3389/fpsyt.2021.734825

**Published:** 2021-09-28

**Authors:** Simona Iodice, Martina Di Paolo, Jennifer Lynn Barkin, Letizia Tarantini, Silvia Grassi, Marta Redaelli, Marta Serati, Virginia Favalli, Luisa Cirella, Valentina Bollati, Massimiliano Buoli

**Affiliations:** ^1^EPIGET LAB, Department of Clinical Sciences and Community Health, University of Milan, Milan, Italy; ^2^Department of Neurosciences and Mental Health, Fondazione IRCCS Ca'Granda Ospedale Maggiore Policlinico, Milan, Italy; ^3^Department of Community Medicine, Mercer University School of Medicine, Macon, GA, United States; ^4^Department of Mental Health, ASST Rhodense, Rho, Italy; ^5^Department of Pathophysiology and Transplantation, University of Milan, Milan, Italy

**Keywords:** perinatal depression, oxytocin, clock genes, epigenetics, DNA methylation, circadian rhythms, pregnancy

## Abstract

**Background:** Perinatal Depression (PD) is a widespread disabling condition that is hypothesized to be associated with abnormalities in circadian rhythms and neuropeptide release including oxytocin (OXT).

**Methods:** Fourty-four pregnant women (28 with PD, and 16 controls) were evaluated through the Edinburgh Postnatal Depression Scale (EPDS), the State/Trait Anxiety Inventory Form Y (STAI-Y), and the Prenatal Attachment Inventory (PAI). A blood sample was collected from all participants, and OXT plasma levels, DNA methylation of clock genes, as well as of FOXp3 and HERV-W were measured. Linear regression analyses were performed to assess the effect of oxytocin on the methylation of selected genes. Continuous ordinal regression models was further applied to see if the score of rating scales was associated to gene methylation, adjusting for oxytocin-methylation interaction.

**Results:** OXT plasma levels were positively associated with CRY1 methylation. Women with higher OXT plasma levels showed an association between higher degree of CRY2 methylation (thus, reduced expression) and lower EPDS (*OR* = 0.21; *P* = 0.043) and STAI-S scores (*OR* = 6.96; *P* = 0.019). Finally, with high OXT levels, hypermethylation of CRY1 was associated to higher scores on the PAI (*OR* = 2.74; *P* = 0.029) while higher methylation of HERV-W related to lower PAI scores (*OR* = 0.273; *P* = 0.019).

**Conclusion:** Our results suggest a possible protective role played by oxytocin in the development of PD by promoting a favorable methylation profile characterized by reduced expression of CRY1 and CRY2. Moreover, oxytocin strengthens the association between maternal prenatal attachment with a favorable pattern of methylation of clock genes and HERV-W, which is essential for pregnancy outcomes.

## Introduction

Perinatal depression (PD), defined as a major depressive episode with an onset during pregnancy or within 4 weeks after delivery, is one of the most common medical complications during pregnancy and postpartum, with 15% of new mothers experiencing at least one major depressive episode during peripartum (1). PD can cause serious consequences for both the mother and the offspring and it has been linked to preterm delivery and low birth weight (2, 3), preeclampsia, maternal suicide and infanticide (4). In addition, perinatal depressive symptoms negatively influence mother-child bonding, which can result in impaired emotional and cognitive development in the child (5). Moreover, it has been noted that maternal biological changes linked to this psychiatric condition can increase vulnerability to psychopathology in offspring via variations to gametes and the gestational uterine environment (6). In light of the significant consequences of untreated PD not only for the health of the mothers, but also of their offspring, the search for reliable biomarkers could be useful for diagnosis and treatment at an early stage and to develop selective targeted therapies to improve the prognosis of these patients.

From this perspective, there has been recently a rising interest in epigenetic biomarkers involved in PD. The focus of epigenetics is the study of changes in gene expression by different mechanisms including DNA methylation and histone modifications (7). Epigenetic changes are a form of adaptive response that allows the genome to prepare for various environmental events and some of these adaptations, which result from early life events, may be persistent, resulting in life-long susceptibility to neuropsychiatric disorders that can be transmitted to subsequent generations, representing a form of “transgenerational transmission” of neuropsychiatric vulnerability (8). For these reasons, in the last years, epigenetics has become an appealing field in the search for new biomarkers for PD, as alterations of biological systems typically involved in the disorder, such as the hypothalamic–pituitary–adrenal axis, the increase in inflammatory response, and the disturbances in circadian rhythms, can be traced back to abnormalities in the expression of corresponding genes (9).

Clock genes play a fundamental role in the regulation of circadian rhythms, which are typically disrupted in Major Depressive Disorder (MDD) and PD, as shown by anomalies in circadian patterns of hormone secretion, body temperature, and cognitive function. However, the sleep wake cycle appears to be the most impaired system (10). Indeed, depressive symptoms were found to be associated with changes in expression of circadian genes (11). In particular, gene *CLOCK* appears to be over expressed in depressed patients compared to controls (12). Increased expression of these genes was associated, in animal models, to an anticipation of circadian rhythms and may therefore account for late insomnia typical of MDD (13). Similarly, PD is characterized by several circadian rhythm perturbations, and animal models suggest that abnormal expression of clock genes can also be observed in physiological pregnancy (14).

Conversely, many other biological functions are altered in both MDD and PD, and recent research emphasizes the role of inflammatory response (15). Data suggest a deep interconnection between the immune and circadian system. On one hand, circadian disruption can lead to dysregulation of immune responses; on the other, inflammation can further disrupt circadian rhythms, as suggested by animal studies that showed that inflammation caused changes in the expression of clock genes by activation of the NF-kB pathway (16, 17). All these observations were reinforced by the results from a recent study by Buoli et al. which demonstrated hypermethylation of *CLOCK* and hypomethylation of *CRY1, PER1, PER2* in depressed pregnant women compared to controls, suggestive of a disruption in circadian rhythms (18). Moreover, the dysregulation of clock genes seemed to coincide with potential immunological abnormalities, as depressed women also showed an increased methylation of gene *FOXp3*, a transcription factor expressed in a subset of CD4+ T cells that modulates the immune system (18).

PD is also characterized by different hormonal alterations and previous studies focused on the role of oxytocin, a neuropeptide traditionally known for its role in stress regulation. Oxytocin (OXT) is known to dampen HPA axis overactivity and for its involvement in delivery and lactation. Additionally, oxytocin increases the salience of social and behavioral cues and contributes to behavioral adaptation to pregnancy and motherhood as maternal OXT functioning influences postpartum maternal attitude and reciprocal parent-infant relationship quality (19). In line with the evidence of its role in modulation of human behavior, low levels of OXT were associated with an increased risk of PD and more severe depressive symptoms (19, 20). In addition, the degree of methylation of the gene for oxytocin receptor (OXTR) was found to be related with the severity of depressive symptoms (21).

Starting from the results of an our previous article (18), which supported the evidence of a disruption in circadian rhythms in depressed pregnant women, the aim of the present study is to investigate the role of oxytocin plasma levels in influencing the degree of methylation of different clock genes (*CLOCK, ARNTL, PER1, PER2, PER3, CRY1, CRY2*) and of other related genes (*OXTR, FOXp3* and *HERV-W*-which encodes for a protein which plays a fundamental role in maternal immune tolerance during pregnancy) in women affected by PD and healthy controls. Because of its beneficial role in mood regulation and pregnancy outcomes, we hypothesized that oxytocin could exert a protective role over affective symptoms.

## Methods

### Sample

This study was conducted at the Department of Neurosciences and Mental Health in collaboration with the Epigenetics and Toxicology Lab, Fondazione IRCCS Policlinico, University of Milan. Data for the analyses of the present paper were available for 44 participants. Sixteen healthy controls and 28 patients with PD (DSM-5 criteria) were recruited by the personnel of our consultancy psychiatry service who were perinatal mental health specialists. All women were 18 years of age or older and were recruited during the third trimester of pregnancy. A detailed study description was given to all interested participants, and an informed written consent was obtained. Subjects were not enrolled if they had a history of psychiatric comorbidity or medical complications which could affect methylation of clock genes, such as autoimmune or endocrine diseases. The study protocol was approved by the local Ethics Committee.

### Assessment

Demographic and clinical information were collected at the time of enrollment through structured interviews. Information on length of gestation and birth weight were collected subsequently. Depressive symptoms were assessed using the Edinburgh Postnatal Depression Scale (EPDS), a 10-item self-report scale that investigates depressive symptoms experienced during the past week and is widely used to screen for depressive symptoms both antenatally and postpartum, with a high grade of reliability (22, 23). A cut-off score of 13 or higher identifies women with clinically significant symptoms (24). Anxiety symptoms were evaluated through the State Anxiety Inventory (STAI), a self report questionnaire that measures the presence and severity of current symptoms of anxiety and a generalized propensity to be anxious (25). Participants also completed the Prenatal Attachment Inventory (PAI), a scale designed to measure prenatal attachment (26). Prenatal attachment defines the special relationship that develops between a mother and her fetus, that is considered fundamental for successful adaptation to pregnancy and for the development of future mother-infant bonding, with implications for child emotional and cognitive development (27).

### Procedures

A single blood sample of 15 mL was obtained from each participant between the 29 and 34th week of gestation. Every sample was collected in late morning to control for diurnal variation in circadian gene expression. Samples were analyzed at the Epigenetics and Toxicology Laboratory, University of Milan. The samples were centrifuged at 1,200 × g for 15 min at room temperature in order to separate plasma, leukocytes and erythrocytes. Oxytocin levels were measured from plasma samples using ELISA technology. Methylation analysis of selected genes were conducted over total leukocytes. Extraction of DNA was performed using Wizard Genomic DNA purification kit (Promega) and total DNA was amplified using Thermo Scientific Nanodrop 1,000 spectrophotometer. Purified DNA was treated with sodium bisulfite and PCR amplification. The treatment with sodium bisulfite converts un-methylated cytosine to uracil while leaving 5-methylcytosine unaltered. Uracil is then converted to thymine upon PCR amplification, thus providing the base to distinguish between methylated and unmethylated CpG sites after PCR, on the basis of percentage of thymine and cytosine. After bisulfite treatment PCR amplification was performed, incorporating a biotinylated primer to separate a single stranded DNA amplicon. The resulting library was sequenced by the pyrosequencing technique (corrected for the percentage of neutrophils) using biotinylated strands as the template for pyrosequencing. Pyrosequencing is a sequencing by synthesis method that quantitatively monitors the incorporations of complementary nucleotides by a DNA polymerase through the enzymatic conversion of released pyrophosphate into a proportional light signal. Light peaks height is proportional to consecutive addition of nucleotides of the same type, thus allowing to quantify the relative abundance of individual nucleotides. Knowing the original genomic sequence of the DNA region in analysis, after bisulfite treatment, the degree of methylation can be determined from the ratio of thymine and cytosine at CpG sites within the amplicon (11).

The degree of methylation of the following genes was analyzed: clock genes (*CLOCK, ARNTL, PER1, PER2, PER3, CRY1, CRY2*), and related genes (*OXTR, FOXp3, and HERV-W*).

### Statistical Analyses

Descriptive analyses on the total sample were performed. For normally distributed demographic and clinical characteristics, data were expressed as mean and standard deviation, otherwise by median and interquartile range. Frequencies and percentages were calculated for categorical variables. The differences between cases and controls were compared using Pearson's chi-square test or Fisher's Exact test for qualitative data, or *T*-test or Mann-Whitney *U*-test, for quantitative variables, as appropriate.

Univariate linear regression analyses were performed to assess the effect of oxytocin on the methylation of selected genes. All methylation genes were natural log-transformed to achieve normality.

Continuous ordinal regression models were applied to assess the association of gene methylation with the rating scale scores (EPDS, STAI, PAI) adjusting for gestational age at enrollment, antidepressants use during pregnancy, and oxytocin-methylation interaction. The effect of methylation on scales was calculated alternatively for low or high oxytocin plasma levels, corresponding to the first-third quartile of its distribution.

We tested for the proportional odds assumption that the effects of the predictors are the same across different levels of the scale scores. The selection of covariates was based on univariate analysis and the best model selection was based on the minimization of the Akaike Information Criterion and maximization of the explained variance of the model.

A two-sided *p* < 0.05 was considered to be significant. Statistical analyses were performed with SAS software (version 9.4).

## Results

The original study sample included 47 eligible pregnant women but three blood samples were not analyzed because of technical problems that interfered with sample processing. Therefore, our final sample was composed by 44 women, 28 of whom matched the criteria for PD and 16 healthy controls.

Demographic and clinical variables of the total sample and of groups are displayed in Table 1. After evaluation, we found no statistically significant differences between depressed and non depressed women with regard to age (*P* = 0.207), gestational age at time of assessment (*P* = 0.331), neonatal birth weight (*P* = 0.141) and other variables that could affect the levels of methylation, such as current smoking (*P* = 0.131) (for further details see Table 1).

**Table 1:** Demographic and clinical characteristics of the 44 pregnant woman.

	**Total sample**	**Healthy**	**Cases**	***P*-value**
	**(*n* = 44)**	**(*n* = 16)**	**(*n* = 28)**	
Maternal age, years, median (Q1–Q3)	35.5 (31, 39)	31.5 (30.5, 40)	36 (33, 39)	0.207
Pre-pregnancy BMI, Kg/m^2^, median (Q1–Q3)	24.2 (21.9, 26.2)	24.2 (22.9, 25.8)	24.8 (21.9, 26.2)	0.952
GA at time of enrollment, weeks, median (Q1–Q3)	32 (30, 35)	33.5 (30.5, 36.5)	32 (30, 35)	0.331
Lenght of gestation, weeks, mean ± SD	39.2 ± 1.4	40 (±1.1)	38.8 (±1.4)	0.012
Infant birth weight, *g*, mean ± SD	3251.9 ± 399.6	3381.9 (±401.3)	3,175 (±387.1)	0.141
History of psychiatric disorder, *n* (%)	18 (41.9%)	1 (6.3%)	17 (60.7%)	0.000
Antidepressants, *n* (%)	19 (51.4%)	0 (0%)	19 (67.9%)	<0.001
Maternal smoke, *n* (%)	10 (21.3%)	1 (6.3%)	9 (32.1%)	0.131
Paternal smoke, *n* (%)	14 (31.8%)	4 (25%)	10 (35.7%)	0.463
Foreign nationality, *n* (%)	8 (17%)	2 (12.5%)	6 (21.4%)	0.697
Oxytocin levels, median (Q1–Q3)	43.7 (38.5, 50.5)	45.9 (38.4, 52.2)	42.6 (38.6, 50.2)	0.724

*GA, gestational age; Q1–Q3, interquartile range*.

Cases and controls only differed in length of gestation. Notably, gestational age at birth was significantly earlier in cases than in controls (38.8 vs. 40 weeks, *P* = 0.01).

### Methylation Analyses

With regard to the effect of oxytocin on methylation of the selected genes, we observed that the neuropetide plasma levels were positively associated with CRY1 methylation levels (Δ% = 3.3; 95% CI: 0.2, 6.5; *P* = 0.040).

With regard the effect of high or low oxytocin plasma levels on the degree of methylation of genes according to rating scale scores, the results show that for high oxytocin levels CRY2 methylation was associated to less severe depressive symptoms (lower EPDS scores) (*OR* = 0.21; *P* = 0.043); this was not verified for lower levels of oxytocin. A similar methylation profile was found for anxiety symptoms, as lower STAI-S scores were associated to higher degree of CRY2 methylation, but only when low levels of oxytocin are present (OR = 6.96; *P* = 0.019); consistently, for high levels of the hormone, increments in CRY2 methylation was associated to a higher risk of more severe anxiety symptoms (higher STAI-S scores) (*OR* = 0.06; *P* = 0.020).

Considering low oxytocin plasma levels, increased oxytocin receptor gene (OXTR) methylation was associated to higher EPDS scores (*OR* = 1.66; *P* = 0.019) and to higher STAI-S scores (*OR* = 1.76; *P* = 0.019).

Finally we found that lower scores on the PAI, reflecting poorer quality of maternal-fetal attachment, were related to higher methylation of HERV-W, but only for high oxytocin levels (*OR* = 0.273; *P* = 0.019). Indeed, when oxytocin levels are higher, we found an association between hypermethylation of CRY1 and higher scores on the PAI (*OR* = 2.74; *P* = 0.029).

## Discussion

To our knowledge this is the first study to investigate the role of oxytocin on methylation patterns of clock genes and the possible association with perinatal depressive symptoms.

Several researches demonstrated a disruption of the circadian clock system in MDD (10, 11), but current literature has a limited number of studies that investigate the relation between clock gene expression during pregnancy and peripartum affective symptoms.

MDD seems to be characterized by an anticipation of circadian rhythms, as suggested by the observation of a typical pattern of altered sleep-wake cycle with late insomnia (28). Alternatively, PD is mainly characterized by initial and central insomnia, with a progressive deterioration of sleep quality in the last trimester of pregnancy and first few weeks after delivery (29). These findings are suggestive of a phase shift in circadian patterns during late pregnancy and postpartum which was demonstrated by recent findings by our group (18).

In our study we examined the supposed disruption of the circadian clock system in PD by analyzing the methylation pattern of circadian genes in depressed and non depressed women. Furthermore, considering the central role played by oxytocin in pregnancy outcomes and regulation of maternal behavior, we hypothesized that oxytocin levels could influence methylation patterns of clock genes, acting as a protective agent against the disruption of circadian rhythms, a symptom frequent in PD. Our hypothesis is consistent with most available data in literature indicating that higher oxytocin plasma levels are associated with lower depressive symptoms during perinatal period, in line with previously documented anxiolytic and antidepressant effects of oxytocin (20). However, no clear mechanism for the role of oxytocin in modulating affective symptoms during perinatal period has been proposed till now.

The main result from our analysis was the observation that high levels of oxytocin seem to improve the methylation profile of depressed patients, given the association between oxytocin plasma levels and reduced expression of CRY1. Animal models showed over-expression of CRY1 in placental and uterine tissues during physiological pregnancy (14) and results from Buoli et al. (18) suggest that depressive and anxiety symptoms, which are associated with gene hypomethylation, can exacerbate a physiological delay of circadian rhythms during pregnancy. Therefore, the observation that oxytocin levels are associated with a higher methylation grade of CRY1, in contrast with the methylation profile described for depressed patients, strongly suggests that oxytocin may regulate maternal mood during pregnancy by normalization of circadian rhythms.

This hypothesis is further supported by results that showed that in women who display high oxytocin levels, reduced expression of CRY2 was associated with less severe depressive symptoms. In our previous study (18) CRY2 was over-expressed in depressed pregnant women; thus the former observation implies that oxytocin may contribute to the maintenance of a more favorable methylation profile, protecting mothers at risk of PD from developing severe symptoms. A similar pattern of methylation was also observed in association with less severe anxiety symptoms, when oxytocin levels were higher.

Finally, in the presence of higher levels of the hormone, a better quality of maternal prenatal attachment (high PAI scores) was associated with a favorable pattern of CRY1 methylation, given the hypo-expression of this gene which, in contrast, is usually over-expressed in depressed pregnant women. These results suggest an association between oxytocin plasma levels and prenatal attachment, which in turn seems to mitigate the disruption of physiological methylation patterns of clock genes. These results are in line with previous studies that demonstrated higher attachment scores in women who displayed increasing oxytocin plasma levels during pregnancy and early postpartum (30). Interestingly, with high oxytocin levels, lower scores on the PAI were associated to lower expression of HERV-W gene, which encodes for an essential protein coopted for human placentation and development of maternal immune tolerance during pregnancy (31). This data is consistent with results from our previous study and suggests that, when HERV-W, which is essential for the positive outcome of pregnancy, is hypo-expressed, maternal antenatal attachment is also deficient. Of note, prenatal attachment not only influences the relationship between the mother and the neonate after delivery, but it is also an independent variable related to favorable neonatal outcomes (27, 32).

## Conclusion

Our results point to the possible protective role played by oxytocin in the development of PD, given its association with a favorable methylation profile of the studied genes which is related to a better functioning of the circadian clock system. Moreover, oxytocin strengthens the association between maternal prenatal attachment with a favorable pattern of methylation, which may account for the positive effect of prenatal attachment on pregnancy outcomes.

Taken together these results suggest that endogenous oxytocin may act as a damper of the detrimental effects of anxiety and depression over the functioning of the circadian clock system and by promoting a better quality of maternal-fetal interaction.

Limitations of the study include the small sample size and that circadian gene methylation profile was assessed in peripheral leukocytes. It is still unclear whether peripheral expression of circadian genes reflect the functioning of the central circadian clock system in the hypothalamus. However, there is evidence that circadian patterns of gene expression are disrupted also at a central level in depressed patients (11), raising the possibility that this disorganization of circadian central oscillators extends to peripheral tissues as well.

Further studies are needed to better understand the mechanisms of interaction between oxytocin levels and gene methylation profile in women affected by PD, but, even so, oxytocin appears to have a clear, although complex, role in the development of affective symptoms during the perinatal period and may be therefore considered for its role in early diagnosis and treatment of PD.

## Data Availability Statement

The raw data supporting the conclusions of this article will be made available by the authors, without undue reservation.

## Ethics Statement

The studies involving human participants were reviewed and approved by Ethics Committee of Fondazione IRCCS Policlinico. The patients/participants provided their written informed consent to participate in this study.

## Author Contributions

SI designed the study and performer statistical analyzes. MD wrote the first draft of the manuscript. JB contributed to study design and reviewed the manuscript. LT performed laboratory analyzes. SG contributed to collect data. MR contributed to sample recruitment and collection of data. MS contributed to study design. VF collected blood samples. LC coordinated the collection of blood samples. VB reviewed the manuscript and coordinated laboratory analyzes. MB wrote the manuscript and contributed to study design and collection of data. All authors contributed to the article and approved the submitted version.

## Conflict of Interest

The authors declare that the research was conducted in the absence of any commercial or financial relationships that could be construed as a potential conflict of interest.

## Publisher's Note

All claims expressed in this article are solely those of the authors and do not necessarily represent those of their affiliated organizations, or those of the publisher, the editors and the reviewers. Any product that may be evaluated in this article, or claim that may be made by its manufacturer, is not guaranteed or endorsed by the publisher.
